# 2092 A multicenter study of fecal microbiota transplantation for Clostridium difficile infection in children

**DOI:** 10.1017/cts.2018.237

**Published:** 2018-11-21

**Authors:** Maribeth R. Nicholson, Erin Alexander, Mark Bartlett, Penny Becker, Zev Davidovics, Elizabeth E. Knackstedt, Michael Docktor, Michael Dole, Grace Felix, Jonathan Gisser, Suchitra Hourigan, Kyle Jensen, Jess Kaplan, Judith Kelsen, Melissa Kennedy, Sahil Khanna, McKenzie Leier, Jeffery Lewis, Ashley Lodarek, Sonia Michail, Paul Mitchell, Maria Oliva‐Hemker, Tiffany Patton, Karen Queliza, Namita Singh, Aliza Solomon, David Suskind, Steven Werlin, Richard Kellermayer, Stacy Kahn

**Affiliations:** 1 Johns Hopkins Children’s Center, Baltimore, MD, USA; 2 Nationwide Children’s Hospital, Columbus, OH, USA; 3 Inova Children’s Hospital, Falls Church, VA, USA; 4 Primary Children’s Hospital at University of Utah, Salt Lake City, UT, USA; 5 MassGeneral Hospital for Children, Boston, MA, USA; 6 Children’s Hospital of Philadelphia, Philadelphia, PA, USA; 7 Mayo Clinic, Rochester, MN, USA; 8 Boston Children’s Hospital, Boston, MA, USA; 9 Children’s Center for Digestive Healthcare at Children’s Healthcare of Atlanta, Atlanta, GA, USA; 10 University of Southern California, Los Angeles, CA, USA; 11 University of Chicago, Chicago, IL, USA; 12 Baylor College of Medicine, Houston, TX, USA; 13 Cedars Sinai Medical Center, Los Angeles, CA, USA; 14 Weill Cornell Medicine, New York, NY, USA; 15 Seattle Children’s Hospital at University of Washington, Seattle, WA, USA; 16 Medical College of Wisconsin, Milwaukee, WI, USA

## Abstract

OBJECTIVES/SPECIFIC AIMS: Clostridium difficile infection (CDI) is the most common cause of antibiotic-associated diarrhea and an increasingly common infection in children in both hospital and community settings. Between 20% and 30% of pediatric patients will have a recurrence of symptoms in the days to weeks following an initial infection. Multiple recurrences have been successfully treated with fecal microbiota transplantation (FMT), though the body of evidence in pediatric patients is limited primarily to case reports and case series. The goal of our study was to better understand practices, success, and safety of FMT in children as well as identify risk factors associated with a failed FMT in our pediatric patients. METHODS/STUDY POPULATION: This multicenter retrospective analysis included 373 patients who underwent FMT for CDI between January 1, 2006 and January 1, 2017 from 18 pediatric centers. Demographics, baseline characteristics, FMT practices, C. difficile outcomes, and post-FMT complications were collected through chart abstraction. Successful FMT was defined as no recurrence of CDI within 60 days after FMT. Of the 373 patients in the cohort, 342 had known outcome data at two months post-FMT and were included in the primary analysis evaluating risk factors for recurrence post-FMT. An additional six patients who underwent FMT for refractory CDI were excluded from the primary analysis. Unadjusted analysis was performed using Wilcoxon rank-sum test, Pearson χ^2^ test, or Fisher exact test where appropriate. Stepwise logistic regression was utilized to determine independent predictors of success. RESULTS/ANTICIPATED RESULTS: The median age of included patients was 10 years (IQR; 3.0, 15.0) and 50% of patients were female. The majority of the cohort was White (89.0%). Comorbidities included 120 patients with inflammatory bowel disease (IBD) and 14 patients who had undergone a solid organ or stem cell transplantation. Of the 336 patients with known outcomes at two months, 272 (81%) had a successful outcome. In the 64 (19%) patients that did have a recurrence, 35 underwent repeat FMT which was successful in 20 of the 35 (57%). The overall success rate of FMT in preventing further episodes of CDI in the cohort with known outcome data was 87%. Unadjusted predictors of a primary FMT response are summarized. Based on stepwise logistic regression modeling, the use of fresh stool, FMT delivery via colonoscopy, the lack of a feeding tube, and a lower number of CDI episodes before undergoing FMT were independently associated with a successful outcome. There were 20 adverse events in the cohort assessed to be related to FMT, 6 of which were felt to be severe. There were no deaths assessed to be related to FMT in the cohort. DISCUSSION/SIGNIFICANCE OF IMPACT: The overall success of FMT in pediatric patients with recurrent or severe CDI is 81% after a single FMT. Children without a feeding tube, who receive an early FMT, FMT with fresh stool, or FMT via colonoscopy are less likely to have a recurrence of CDI in the 2 months following FMT. This is the first large study of FMT for CDI in a pediatric cohort. These findings, if confirmed by additional prospective studies, will support alterations in the practice of FMT in children.

